# miR394 and LCR are involved in Arabidopsis salt and drought stress responses in an abscisic acid-dependent manner

**DOI:** 10.1186/1471-2229-13-210

**Published:** 2013-12-11

**Authors:** Jian Bo Song, Shuai Gao, Di Sun, Hua Li, Xia Xia Shu, Zhi Min Yang

**Affiliations:** 1 Weigang No. 1, College of Life Science, Nanjing Agricultural University, Nanjing 210095, China

**Keywords:** miR394, LCR, Abscisic acid, Salinity, Drought, Arabidopsis

## Abstract

**Background:**

MicroRNAs (miRNAs) are a class of short, endogenous non-coding small RNAs that have ability to base pair with their target mRNAs to induce their degradation in plants. miR394a/b are conserved small RNAs and its target gene *LCR* (*LEAF CURLING RESPONSIVENESS*) encodes an F-box protein (*S*KP1-*C*ullin/*C*DC53-*F*-box) but whether miR394a/b and its target gene *LCR* are involved in regulation of plant response to abscisic acid (ABA) and abiotic stresses is unknown.

**Results:**

Mature miR394 and precursor miR394a/b are shown to be slightly induced by ABA. By contrast, *LCR* expression is depressed by ABA. Analysis of *LCR* and its *promoter* (*pLCR::GUS*) revealed that *LCR* is expressed at all development stages. *MIR394a/b* over-expression (*35S::MIR394a/b*) and *lcr* (*LCR* loss of function) mutant plants are hypersensitive to salt stress, but *LCR* over-expressing (*35S::m5LCR*) plants display the salt-tolerant phenotype. Both *35S::MIR394a/b* and *lcr* plants are highly tolerant to severe drought stress compared with wild-type, but *35S::m5LCR* plants are susceptible to water deficiency. Over-expression of *MIR394a/b* led to ABA hypersensitivity and ABA-associated phenotypes, whereas *35S::m5LCR* plants show ABA resistance phenotypes. Moreover, *35S::MIR394a/b* plants accumulated higher levels of ABA-induced hydrogen peroxide and superoxide anion radicals than wild-type and *35S::m5LCR* plants. Expressions of ABA- and stress-responsive genes, *ABI3*, *ABI4*, *ABI5*, *ABF3*, and *ABF4* are up-regulated in *MIR394a/b* over-expressing plants but down-regulated in *35S::m5LCR* plants. Over-expression of *MIR394a* in *abi4-1* or *abi5-1* background resulted in loss of ABA-sensitivity in *35S::MIR394a* plants.

**Conclusions:**

The silencing of *LCR* mRNA by miR394 is essential to maintain a certain phenotype favorable for the adaptive response to abiotic stresses. The contrasting phenotypes of salt and drought responses may be mediated by a functional balance between miR394 and *LCR*. If the balance is perturbed in case of the abiotic stress, an identical phenotype related to the stress response occurs, resulting in either ABA sensitive or insensitive response. Thus, miR394-regulated LCR abundance may allow plants to fine-tune their responses to ABA and abiotic stress.

## Background

Salinity and drought are one of the major environmental stresses that limit worldwide plant growth and crop production. Plants have evolved multiple sophisticated strategies to cope with the adverse stresses via perceiving the stress signal and transmitting the information through a variety of signal transduction pathways and adjustment of their metabolic processes; upon receipt of the signal, a number of molecular and cellular responses are initiated [1]. The ABA (abscisic acid)-based complex signal transduction cascades are critical for plant adaptation to environmental stresses [2,3]. Recent studies have identified many components acting between ABA perception and abiotic stress responses, including ABA biosynthetic enzymes and regulatory intermediates such as kinases, transcription factors and ubiquitin ligases [4]. Among these, E3 ubiqutin ligases were reported to participate in activation of ABA response and degradation of signaling components associated with the stress responses [5]. ABI5 (ABA insensitive 5) is a member of Arabidopsis basic leucine zipper (bZIP) transcription factor family that inhibits ABA-dependent seed germination and post-germination growth [6]. KEG is a multi-domain RING-type E3 ligase required for maintaining low levels of ABI5 in the absence of ABA [7]. Whereas over-expression of *KEG* leads to ABA insensitivity, disruption of *KEG* gene expression results in growth arrest immediately after seed germination.

Recently, the post-transcriptional regulation of ABA- and stress-responsive genes by a group of miRNAs has received much attention [8-10]. Over-expressing miR396c conferred sensitivity to salinity and alkaline stress [11]. The stress-regulated miR393-guided cleavage of transcripts encoding two auxin receptors, TIR1 and AFB2, is necessary for inhibition of lateral root growth under ABA treatment and osmotic stress [12]. miR168a over-expressing and AGO1 loss-of-function *ago1-27* mutant plants display ABA hypersensitivity and drought tolerance, whereas *mir168a-2* mutant plants show ABA hyposensitivity and drought hypersensitivity [13]. However, conflicting reports exist on miRNAs regulation of plant abiotic stress responses. For example, AtmiR169 targets a gene coding for a ubiquitous transcription factor NFYA5; over-expression of AtmiR169a in plants enhanced leaf water loss and more sensitivity to drought stress than wild-type plants [14], whereas Sly-miR169c over-expression enhanced drought tolerance by reducing stomatal opening, transpiration rate and leaf desiccation [15]. These results suggest that miRNAs-regulated plant response to abiotic stresses seems more complex than expected. Their biological roles and regulatory networks that coordinate plant response to abiotic stresses are still not fullly understood.

miR394 is one of the conserved miRNAs that exist in many dicot and monocot plant species; in Arabidopsis, only two miR394a and miR394b were found and its target gene (*At1g27340*) was found to encode an F-box protein (*S*KP1-*C*ullin/*C*DC53-*F*-box) [8]. Recently, we have reported miR394 and its target gene *LCR* are involved in regulation of leaf curling-related morphology [16]. Over-expression of a miR394-resistant version of *LCR* under the 35S promoter (*35S:m5LCR*) results in a curled-down leaf defect. Conversely, transgenic plants over-expressing *35S:MIR394a/b* display a curled-up leaf phenotype. Detailed analysis showed evidence that these phenotypes are related to auxin response. Furthermore, miR394 has been identified as a mobile signal on the surface cell layer (the protoderm) required for enhancing stem cell competence to the distal meristem by repressing LCR [17]. Interestingly, miR394 was also reported to be induced by abiotic stresses in plants [8,18-22]. These findings prompted us to investigate further whether miR394 is able regulate plant responses to salt and drought stresses. The present study provides evidence that miR394 is involved in regulation of plant response to salt and drought stresses in Arabidopsis in an ABA-dependent manner.

## Results

### Expression of miR394 under salt and drought stresses and ABA treatment

**Figure 1:**
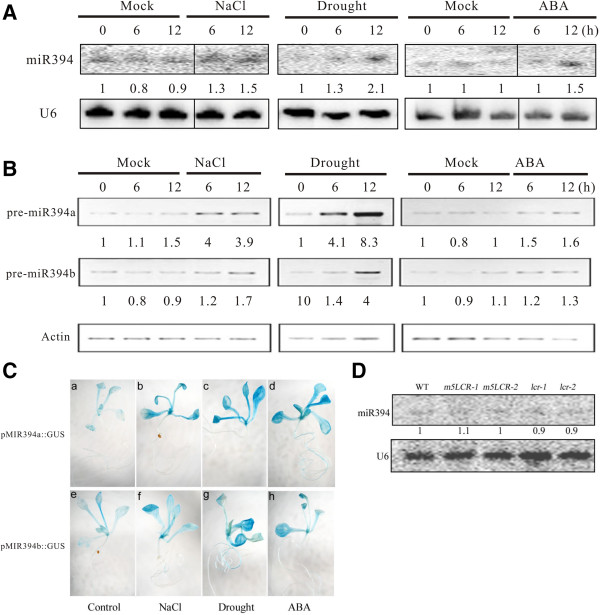
**Transcriptional expression of mature miR394, precursor miR394a/b (pre-miR394a/b), and *pMIR394::GUS *staining in the wild-type or transgenic Arabidopsis carrying *pMIR394a/b::GUS *under salt, desiccation and ABA treatment.** Two week-old wild-type Arabidopsis seedlings were treated with NaCl (300 mM), desiccation, and ABA (100 μM) for 0, 6 and 12 h. The expression data were obtained using Northern blot **(A)** and RT-PCR **(B)** analysis. The number below the band indicates that the value for 0 h is considered as 1.0, on which the treatments were normalized. **(C)**: GUS staining in two week-old *pMIR394a/b::GUS* transgenic seedlings treated with salt (300 mM NaCl) (b, f), drought (c, g) and 100 μM ABA (d, h) for 12 h. **(D)**: RNA gel blot analysis of miR394 expression in the wild-type, *35S::m5LCR-1*, *35S::m5LCR-2*, *lcr-1* and *lcr-2*.

To validate expression of miR394 in response to salt and drought stresses, two week-old Arabidopsis seedlings were exposed to 300 mM salt (NaCl), drought (desiccation) and 100 μM ABA for 6 and 12 h. RNA gel-blot analysis showed that miR394 was slightly induced by NaCl and drought (Figure 1A). Using RT-PCR, we further analyzed whether expression of miR394a and miR394b was also affected by salt and drought exposure. It is shown that both miR394a and miR394b were induced under saline and drought stresses (Figure 1B; Additional file 1: Figure S1). To further investigate expression of miR394a/b, its promoter sequences were retrieved, fused to the *β-glucuronidase* (*GUS*) reporter gene, and introduced into transgenic plants. The histochemical analysis showed that heavy GUS staining was detected in *pMIR394a/b::GUS* seedlings under salt and drought stresses, whereas the relatively light GUS staining was observed in the control seedlings (Figure 1C). The phytohormone ABA mediates plant response to abiotic stresses [1]. Examination of miR394 response to ABA treatment revealed that miR394 was mildly induced (Figure 1A). Similarly, the abundance of both *pre-miR394a* and *pre-miR394b* transcripts as well as *pMIR394a/b::GUS* staining were also slightly enhanced by ABA (Figure 1B,C; Additional file 1: Figure S1). Feedback loops in which miRNA-regulated genes may regulate the transcription of their miRNA have been described before [23]. Our analysis showed that miR394 transcripts were not changed in the *35S:mLCR* and *lcr* mutant plants (Figure 1D); also, quantitative real-time PCR showed no significant deference between *35S:mLCR* or *lcr* mutant plants and wild-type (Additional file 1: Figure S2), indicating that miR394 is not feedback-regulated by *LCR*.

### Expression of LCR under salt and drought stresses and ABA treatment

**Figure 2:**
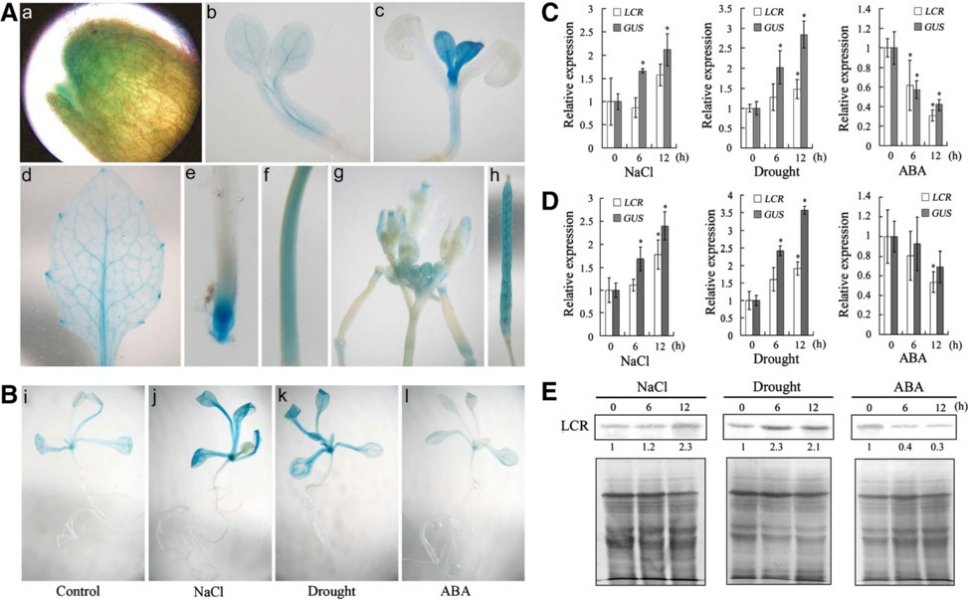
**Expression of *LCR *(*at1g27340*) in developing Arabidopsis and its regulation by abiotic stresses and ABA treatment. A**: *pLCR::GUS* expression pattern in different tissues: GUS expression in germinating seeds (a); cotyledons of four day-old seedlings (b); true leaves of seven day-old seedlings (c); rosette leaves of the thirty day-old plants (d); root tips of fourteen day-old seedlings (e); stems (f); flowering tissues (g); and siliques (h). **B**: GUS staining in two week-old *pLCR::GUS* transgenic seedlings treated with salt (300 mM NaCl) (j), drought (k) and 100 μM ABA (l) for 12 h. **C/D/E**: Real time qRT-PCR and Western-bolt analyses on expression of RNA level of *LCR* and *GUS* in *pLCR::GUS* plants or protein level of LCR in wild-type. Two week-old wild-type Arabidopsis seedlings were treated with NaCl (300 mM), and ABA (100 μM) for 6–12 h. The data were obtained from qRT-PCR (**C**: *pLCR::GUS* line-1; **D**: *pLCR::GUS* line-2) and Western-bolt analysis **(E)**. Vertical bars represent standard deviation of the mean (*n* = 3, treatments with 60 seedlings). Asterisks indicate the significant difference between the treatments and control (*P*<0.05).

Although miR394 is induced by salt and drought, whether LCR is regulated by ABA and the abiotic stresses is unknown. To investigate the expression pattern of *LCR*, we generated constructs encoding a GUS reporter protein and *LCR* fusion protein under the control of *LCR* promoter sequence (1.45 kb) and transformed into Arabidopsis. Histochemical staining showed that *pLCR::GUS* was active at all development stages (Figure 2A). To test the regulation of *LCR* promoter by abiotic stresses, two week-old seedlings were exposed to NaCl, drought and ABA. Treatments with salt and drought induced higher GUS activity in *pLCR::GUS* plants compared with the control (Figure 2B). To confirm the expression pattern of GUS staining under the abiotic stresses, we comparatively analyzed the transcripts of *GUS* in *pLCR::GUS* plants and *LCR* in wild-type using qRT-PCR. As shown in Figure 2C,D, the transcript levels of *GUS* and *LCR* were up-regulated 6 and 12 h after NaCl and drought treatments. But the *GUS* level was higher than the *LCR* level. This suggests that LCR mRNA was partially silenced by miR394 in plants under the stress condition. To investigate whether LCR at a translational level was affected by salt and drought, a western blot study was carried out. Using a monoclonal antibody specifically interacting with LCR protein (52 kDa), we showed that the LCR proteins could be also induced by salt and drought treatments (Figure 2E). Unexpectedly, less GUS staining was detected in seedlings with ABA treatment (Figure 2B). Also, both LCR transcript and protein abundance with ABA was found to be lower than the control (Figure 2C,D,E).

### Identification of *35S::MIR394*, *35S::m5LCR* and *lcr* mutant lines

**Figure 3:**
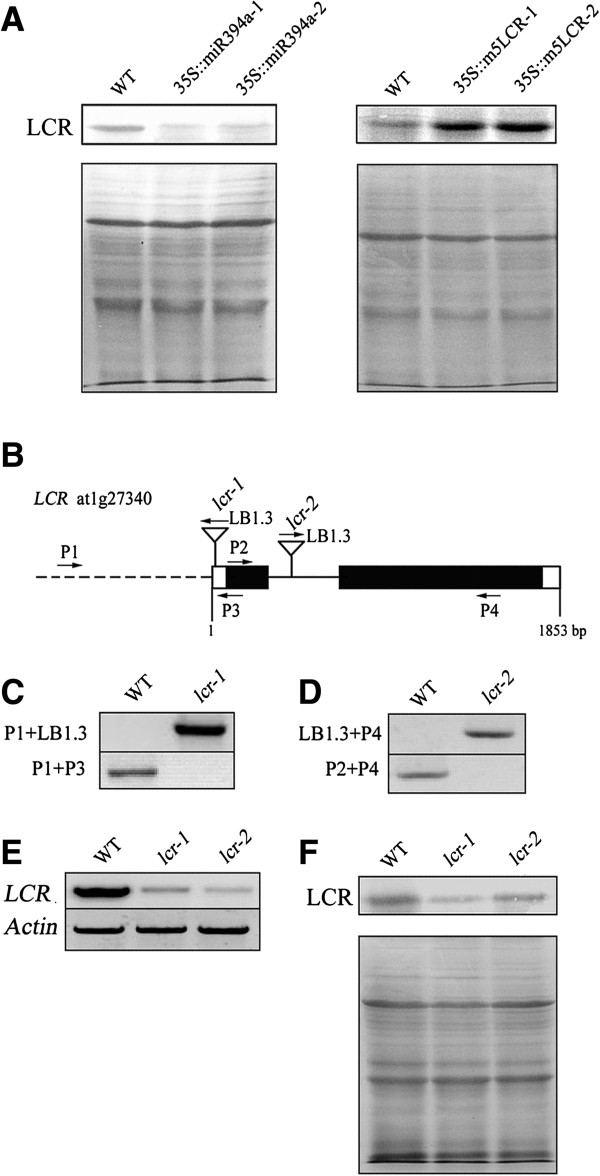
**Characterization of transgenic plants and *lcr *mutant lines. (A)** Assessment of LCR protein in plants grown on MS medium for 14 d. **(B)** Schematic diagram of LCR structure and T-DNA diagnostic PCR and RT-PCR. Closed boxes represent exons, opened boxes represent the 5′ and 3 UTR, solid line between closed boxes represents introns and dash line represents promoter region. Gene-specific (forward and reverse) and T-DNA–specific (LB1.3) primers used in the genotyping and RT-PCR are shown with arrows. **(C)** and **(D)** Diagnostic PCR of T-DNA inserted in the two different regions of LCR. DNA from insertion lines of *lcr-1* (SALK_016763c) and *lcr-2* (SALK_136833c) were used. **(E)** RT-PCR analysis of the LCR transcripts in wild-type and T-DNA insertion mutant seedlings. **(F)** Detection of the LCR protein in wild-type and *lcr* seedlings.

To identify the role of miR394 in the stress responses, we examined the transgenic lines over-expressing *MIR394a/b* (*35S::MIR394a/b*) and the cleavage-resistant version (*35S::m5LCR*) [16]. The Western blotting on LCR proteins was analyzed. The abundance of LCR proteins was lower in *35S::MIR394a* plants but was higher in *35S::m5LCR* plants compared with the wild-type (Figure 3A), indicating that miR394 affects the abundance of LCR proteins.

The genomic sequence of *LCR* contains two exons (1404 bp), which are interrupted by an intron towards 5′ end; the CDS contains an open reading frame coding for a protein with 467 amino acid residues (Figure 3B). Two independent T-DNA insertion lines, *lcr-1* (SALK_016763c) and *lcr-2* (SALK_136833c) were used. The mutant *lcr-1* has a T-DNA insertion at 5′ UTR, whereas *lcr-2* has an insertion in the intron towards the first exon. The two mutants were verified by diagnostic PCR using LCR gene-specific and T-DNA border primers (Figure 3C,D). The *lcr-1* and *lcr-2* alleles were confirmed by RT-PCR and Western blot, by which the expression of both LCR mRNA and proteins were severely suppressed (Figure 3E,F).

### MIR394a over-expressing and *lcr* mutant plants display salt sensitivity

**Figure 4:**
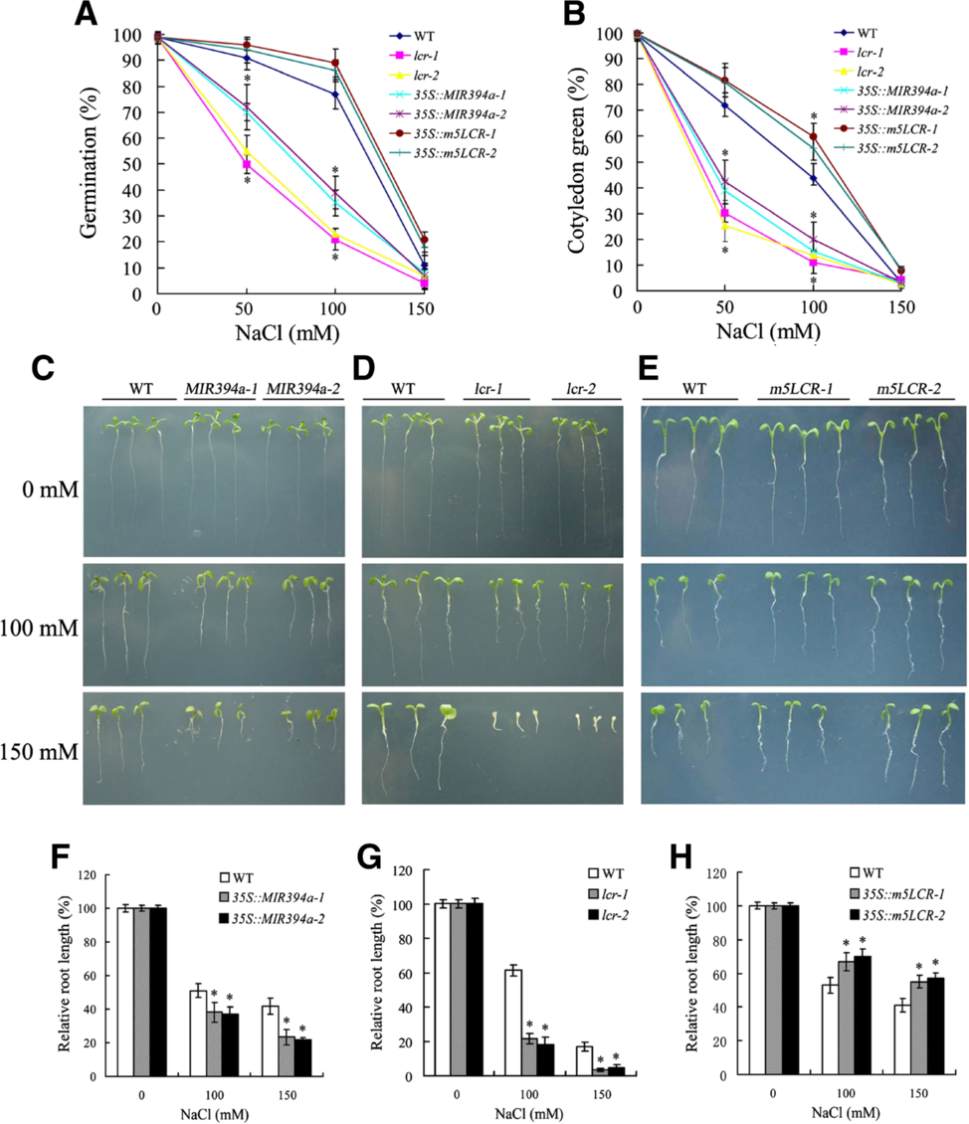
**Germination and growth responses of transgenic plants to salt stress. A** and **B**: Seeds of wild-type, *lcr*, *35S:MIR394a* and *35S:m5LCR* plants were germinated on the plates containing the different concentrations of NaCl. Germination rates **(A)** were measured after 4 d and cotyledon greening **(B)** were measured after 7 d. **(C/D/E)**: Root growth of transgenic plants on MS medium with 100 and 150 mM NaCl. Seeds were germinated for 10 of **(C)**, 10 **(D)** and 7 **(E)** d, respectively, on the MS medium with or without NaCl. F/G/H: Root length of the indicated genotypes grown on medium with different concentrations of NaCl was measured at 10 d **(F/G)**, and 7 d **(H)**, respectively. Relative root growth compared with that on NaCl-free medium is indicated. For *35S:MIR394b* plants, similar results were found (data not shown). Vertical bars represent standard deviation of the mean (*n* =3, treatments with 60 seedlings). Asterisks indicate the significant difference between the transgenic lines/mutants and wild-type (WT) (*P*<0.05).

Plant seeds were placed on the solid MS medium supplemented with 0–150 mM NaCl. The seed germination and post-germination growth responded differently to 50–100 mM NaCl after 4 d treatment (Figure 4A). Following the treatment with 100 mM NaCl, the germination percentages of *35S::MIR394a-1*, *35S::MIR394a-2*, *lcr-1* and *lcr-2* seeds were only 10-20%, whereas those of wild-type were 78%. Simultaneously, the cotyledon greening was blocked by 50 or 100 mM NaCl in *35S::MIR394a* or *lcr* plants. Under the same condition, the germinating rate of *35S::m5LCR* seeds was up to 94%, and the percentage of cotyledon greening in *35S::m5LCR* seedlings was higher than that of wild-type (Figure 4B). Root growth of *35S::MIR394a* and *lcr* seedlings was strongly inhibited by 50–100 mM NaCl relative to the wild-type, but compared with *35S::MIR394a*, the root growth of *lcr* seedlings was more severely inhibited (Figure 4C,D,F,G). By contrast, *35S::m5LCR plants* had higher tolerance of roots to NaCl stress. The root elongation of *35S::m5LCR* plants with 150 mM NaCl was increased by 30-40% compared with wild type (Figure 4E,H). These results indicate that both *lcr* mutant and *35S::MIR394a* plants are hypersensitive, whereas *35S::m5LCR plants* are tolerant to the salt stress.

### MIR394a over-expression and *lcr* mutant plants display drought tolerance

**Figure 5:**
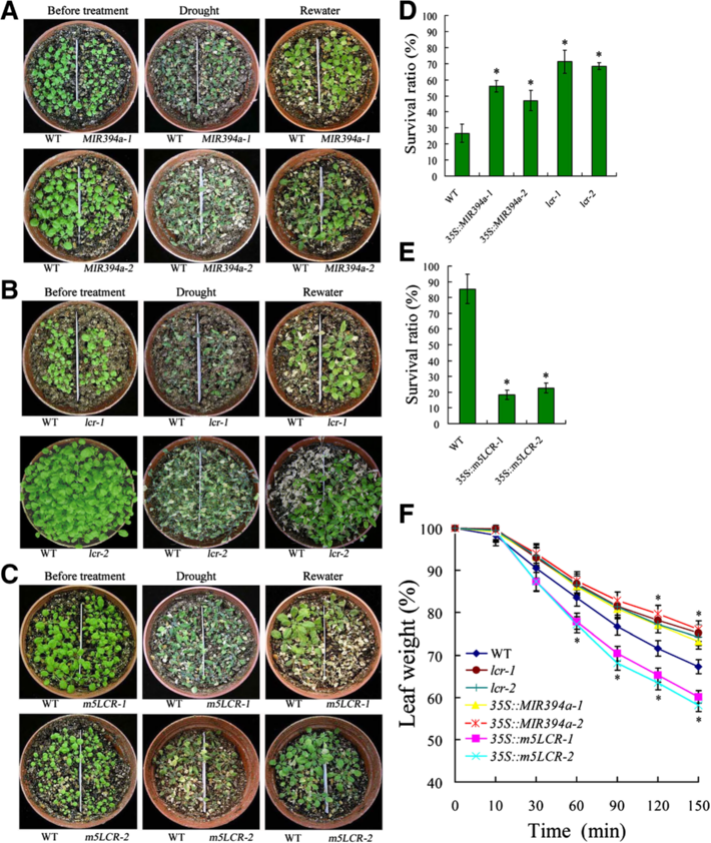
**Responses of transgenic plants to drought. A/B/C**: Three week-old wild-type, *35S::MIR394a***(A)**, *lcr* mutant **(B)** and *35S::m5LCR***(C)** plants were dehydrated for 14 **(A and B)** or 12 **(C)** d, followed by rewatering for 3 d. Dehydration tolerance was assayed as the ability of plants to resume growth when returned to normal conditions. **D**: Survival of wild-type, *35S::MIR394a* and *lcr* mutant plants by rewatering for 3 d after dehydration treatment for 14 d. **E**: Survival of wild-type and *35S::m5LCR* plants by rewatering for 3 d after dehydration treatment for 12 d. **F**: Measurement of water loss rates. Detached rosette leaves of the indicated plants were placed on open-lid Petri-dishes and weighed at the indicated times after excision. Water loss was calculated as the percentage of initial fresh weight. For *35S:MIR394b* plants, similar results were found (data not shown). Vertical bars represent standard deviation of the mean (*n* = 3, treatments with 60–100 seedlings). Asterisks indicate the significant difference between the transgenic lines/mutants and wild-type (WT) (*P*<0.05).

Under the water loss condition, most of wild-type, *35S::MIR394a* and mutant plants were withered (Figure 5A,B). After a three-day re-watering, 47-56% of *35S::MIR394a* and 68-71% of the *lcr* mutant plants resumed their growth, whereas only 26% of the wild-type plants survived (Figure 5D). By contrast, the *35S::m5LCR* plants were severely wilted after dehydration stress (Figure 5C). After re-watering for 3 d, there was only 18-23% survival (Figure 5E), indicating that the *35S::m5LCR* plants are supersensitive to drought stress.

We further examined plant response to drought stress using detached rosette leaves of two week-old seedlings, which were placed on a open-lid petri dish under dim light at room temperature. The reduced fresh weight of leaves was measured over time (0–150 min). Leaves from all leaves showed progressive loss of weight (Figure 5F). To the end of experiment, the fresh weight for *35S::m5LCR* plants was only 58-60% of their starting value, and the fresh weight for *35S::MIR394a* and *lcr* mutant plants was 73-78%. The wild-type had an intermediate weight of 67%. From these results it is shown that while *35S::m5LCR* plants were hypersensitive to water loss, *35S::MIR394a* and *lcr* mutant plants displayed higher tolerance to drought stress.

### MIR394a and LCR are involved in ABA-dependent seed germination and root growth

**Figure 6:**
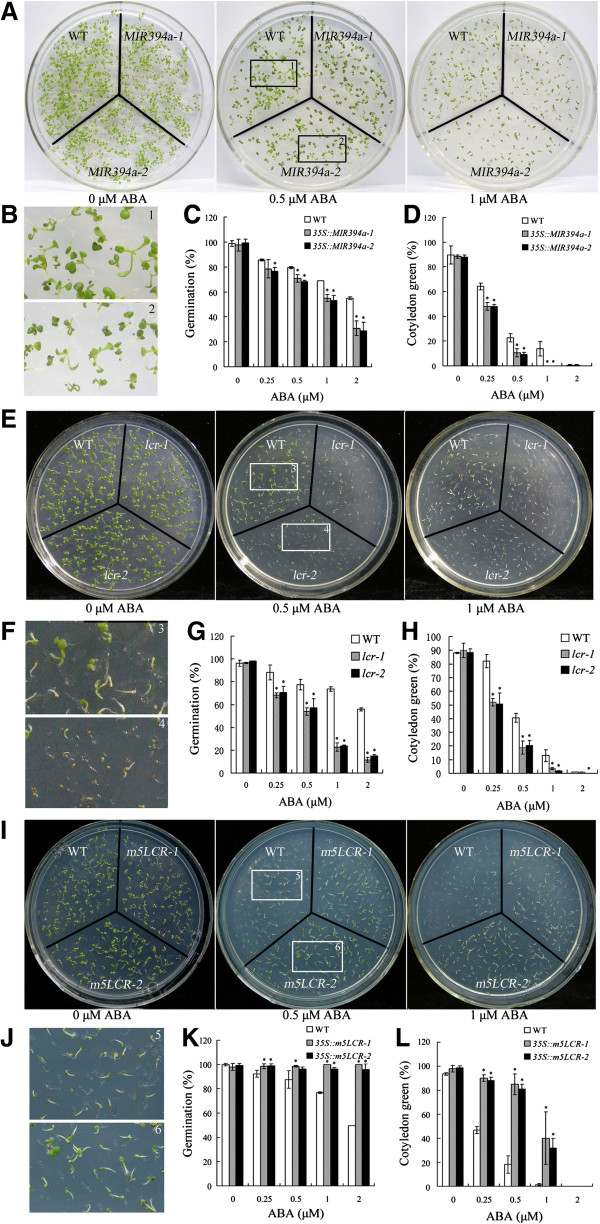
**Response of seed germination and cotyledon growth of transgenic and *lcr *plants to ABA. A/B/, E/F, I/J**: Growth of wild-type, *35S::MIR394a***(A/B)**, *lcr***(E/F)** and *35S::m5LCR***(I/J)** plants on MS medium with 0.5 and 1 μM ABA. Seeds were germinated and grown for 10 d (*35S::MIR394a* and *lcr* plants), and 7 d (*35S::m5LCR* plants). **C/D, G/H, K/L**: ABA-dependent seed germination **(C/G/K)** and cotyledon greening **(D/H/L)**. Seeds of wild-type, *35S::MIR394a***(C/D)**, *lcr***(G/H)** and *35S::m5LCR***(K/L)** plants were germinated plates containing 0.25-2 μM ABA. Seed germination were determined after 4 d and cotyledon greening were determined after 7 d. For *35S:MIR394b* plants, similar results were found (data not shown). Vertical bars represent standard deviation of the mean (*n* =3, treatments with 60–120 seedlings). Asterisks indicate the significant difference between the transgenic lines/mutants and wild-type (WT) (*P*<0.05).

For *35S::MIR394a* and *lcr* plants, seed germination and cotyledon development were severely inhibited by 0.25-2 μM ABA (Figure 6A-H), but seed germination of *35S::m5LCR* plants was unaffected by ABA (even at 2 μM) (Figure 6I-K). Nevertheless, 81-85% of *35S::m5LCR* seedlings were able to develop true green cotyledons in the presence of 0.5 μM ABA, with only 18% of wild-type was presented (Figure 6L). A concentration-dependent change was observed, in which the ABA-hypersensitive response of *35S::MIR394a* and *lcr* plants occurred at 0.25 μM of ABA, and when ABA concentrations were up to 1 μM, the cotyledon growth was completely blocked (Figure 6D,H). By contrast, 35-40% of *35S::m5LCR* seedling cotyledons were still green and even expanded at 1 μM of ABA, where the growth of wild-type was arrested (Figure 6L).

**Figure 7:**
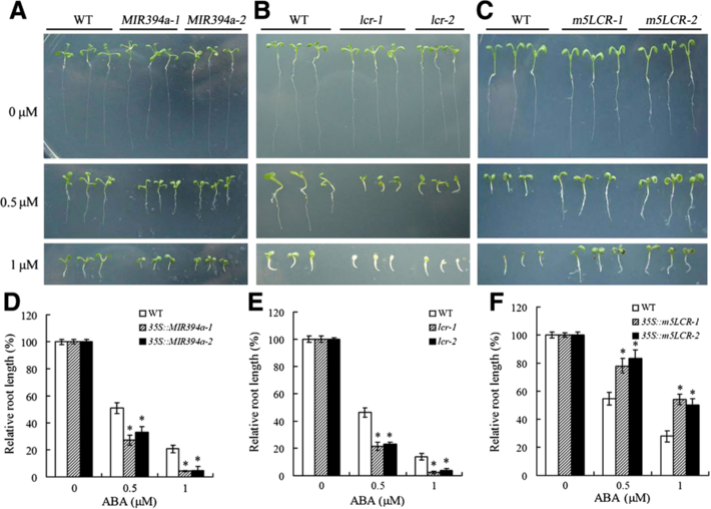
**Response of root growth of transgenic and *lcr *plants to ABA treatment. A/B/C**: Phenotype of root growth of wild-type, *35S::MIR394a*, *lcr* and *35S::m5LCR* plants treated with ABA. Germinated seeds were grown on MS medium containing ABA at 0.5 and 1 μM for 10 (*35S::MIR394a* and *lcr* plants) and 7 (*35S::m5LCR* plants) d. **D/E/F**: Root elongation measurements. Seedling root length of the indicated plants grown on medium with ABA at 0.5 and 1 μM was measured 10 **(D/E)** or 7 **(F)** days after treatment. For *35S:MIR394b* plants, similar resuls were found (data not shown). Vertical bars represent standard deviation of the mean (*n* =3, treatments with 60 seedlings). Asterisks indicate the significant difference between the transgenic lines/mutants and wild-type (WT) (*P*<0.05).

Consistent with seed germination and cotyledon growth, root elongation of *35S::MIR394a* and *lcr* seedlings was hypersensitive to ABA (Figure 7A,B). The sensitive concentration occurred at 0.5 μM, where the root length of *35S::MIR394a* and *lcr* was only 52.9-64.8% and 46.1-49.8% of wild-type, respectively (Figure 7D,E). Under the same condition, however, the elongation of *35S::m5LCR* root showed more resistance to ABA treatment (Figure 7C). At 0.5 and 1 μM of ABA, the root length of *35S::m5LCR* plants was 1.44-1.54 and 1.81-1.95 folds over the wild-type, respectively (Figure 7F). We generated transgenic lines over-expressing a modified version of *IPS1* (*INDUCED BY PHOSPHATE STARVATION 1*), which encodes a non-coding RNA [24]. This RNA comprises a short motif highly complementary to miR394 with a small loop at the expected cleavage position, which can sequester miR394, partially release its natural target, and increase LCR abundance [16]. We used target mimicry *miR394* (*MIM394*) transgenic lines to test ABA response in plants and found that phenotype of root growth is very similar to that of *35S::m5LCR* plants (Additional file 1: Figure S3). Thus, *MIR394* over-expressing plants were hypersensitive to ABA, whereas *35S::m5LCR* plants had a converse phenotype with ABA.

### MIR394a and LCR are involved in ABA-dependent stomatal closure

**Figure 8:**
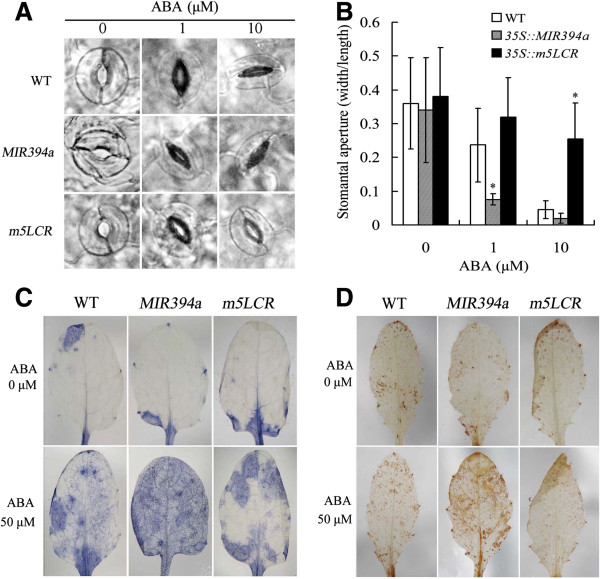
**Stomatal aperture and reactive oxygen species production in leaves of transgenic plants with ABA. A**: ABA-induced stomata closure. Four week-old mature leaves of wild-type (WT), *35S::MIR394a* and *35S::m5LCR* plants were incubated in stomatal opening solution for 2 h and transferred to solutions containing the indicated concentrations of ABA for 2 h. Stomata on the abaxial surface were observed using light microscopy. **B**: Measurement of stomatal aperture after ABA treatment. At least 60 stomatal cells from each sample were measured. **C** and **D**: Detection of ABA-induced O_2_^–^ and H_2_O_2_ accumulation in the leaves of the plants. Leaves from four week-old plants were treated with 0 or 50 μM ABA for 2 h and transferred to 0.1 mg mL^-1^ nitroblue tetrazolium **(C)** and 0.1 mg mL^-1^ 3,3’-diaminobenzidine **(D)** solution for 2 h. The color representing the O_2_^–^ and H_2_O_2_ abundance in the leaves were visualized and photographed. For *35S:MIR394b* plants, similar results were found (data not shown). Vertical bars represent standard deviation of the mean (*n* =3, treatments with 60 seedlings). Asterisks indicate the significant difference between the transgenic lines/mutants and wild-type (WT) (*P*<0.05).

Leaves of 4 week-old plants were submerged in stomatal opening solution and treated with ABA at 0, 1, and 10 μM for 2 h. In the absence of ABA, all guard cells on leaves of plants were fully opened (Figure 8A). When 1 and 10 μM ABA was added to the solution, the stomata on the leaves of wild-type and *35S::MIR394a* plants were closed. Quantitative analysis using stomatal aperture (the ratio of width to length) showed that there was much stronger stomatal closure in *35S::MIR394a* plants than in wild-type with 1 μM ABA (Figure 8B). Conversely, the *35S::m5LCR* leaves were insensitive to ABA-induced stomatal closure and most of guard cells were open in the presence of 1 or 10 μM ABA. The stomatal apertures were 1.4- (1 μM ABA) and 5.6- (10 μM ABA) fold over those of wild-type, respectively.

Reactive oxygen species (ROS) is one of the essential signal molecules involved in abscisic acid (ABA)-induced stomatal closure [25]. Leaves of four week-old plants were exposed to 0 or 50 μM ABA for 2 h and treated with nitroblue tetrazolium (NBT) or 3,3’-diaminob enzidine (DAB). Compared with wild-type, leaves of *35S::MIR394a* plants exposed to 50 μM ABA were stained intensively, whereas those of *35S::m5LCR* plants were stained light with O_2_^–.^ and H_2_O_2_ (Figure 8C,D). These results indicated that ABA-induced ROS abundance could be altered in *35S::MIR394a* and *35S::m5LCR* plants.

### Over-expression of miR394a cannot rescue the ABA insensitivity of *abi4-1* and *abi5-1*

**Figure 9:**
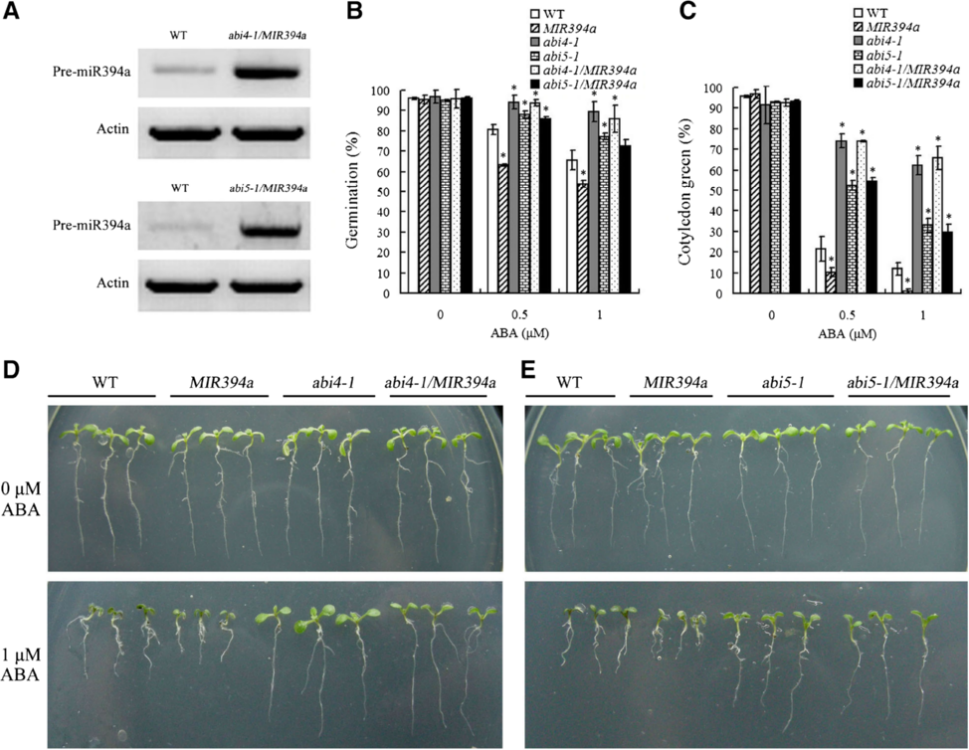
**Phenotypical analysis of the cross lines of *abi4-1/MIR394a *(or *abi4-1/35S::MIR394a*) and *abi5-1/MIR394a *(or *abi5-1/35S::MIR394a*) with ABA. A**: RT-PCR analysis of in *pre-miR394a* expression in the two week-old *abi4-1/MIR394a* and *abi5-1/MIR394a* plants. **B/C**: Analysis of germination rates and cotyledon greening. Seeds of the indicated plants were germinated and grown for 4 **(B)** or 7 **(C)** d on plates containing 0, 0.5 and 1 μM ABA. After that, seed germination and cotyledon greening were determined. **D/E**: Root growth of the indicated plants on the MS medium with 0 or 1 μM ABA. The phenotypes were photographed 10 d after germination. For *35S:MIR394b* plants, similar results were found (data not shown). Vertical bars represent standard deviation of the mean (*n* =3, treatments with 60 seedlings). Asterisks indicate the significant difference between the transgenic lines/mutants and wild-type (WT) (*P*<0.05).

To gain insights into involvement of miR394 in ABA signaling pathway, *abi4-1* and *abi5-1* mutant plants were individually crossed to the *35S::MIR394a* plants. Both *ABA-INSENSITIVE* (*ABI*) *4* and *ABI5* encode the major transcription factors which act as positive regulators of ABA response [4,7]. Because *35S::MIR394a* and *abi4-1* or *abi5-1* mutant plants display the opposite phenotypes, analysis of the crossed plants can elucidate the relationship between the genes. *preMIR394a* were expressed in the *abi4-1/35S::MIR394a* and *abi5-1/35S::MIR394a* plants (Figure 9A). Examination of the two cross plants revealed that the phenotypes of *abi4-1/35S::MIR394a* and *abi5-1/35S::MIR394a* plants were nearly identical to the phenotype of *abi4-1* and *abi5-1* plants, respectively [6,26,27]. In the presence of 0.5 or 1 μM ABA, both *abi4-1/35S::MIR394a* and *abi5-1/35S::MIR394a* plants showed higher seed germination rates, enhanced cotyledon development, and increased root elongation (Figure 9B-E). These results indicate that over-expression of *MIR394a* in *abi4-1* or *abi5-1* background resulted in loss of ABA-sensitivity in *35S::MIR394a* plants. The data suggest that miR394a does not function in parallel to ABI4 and ABI5, but both ABI4 and ABI5 may work downstream of miR394a*.*

### Expression of ABA and stress responsive genes in *35S::miR394a* and *35S::m5LCR* plants

**Figure 10:**
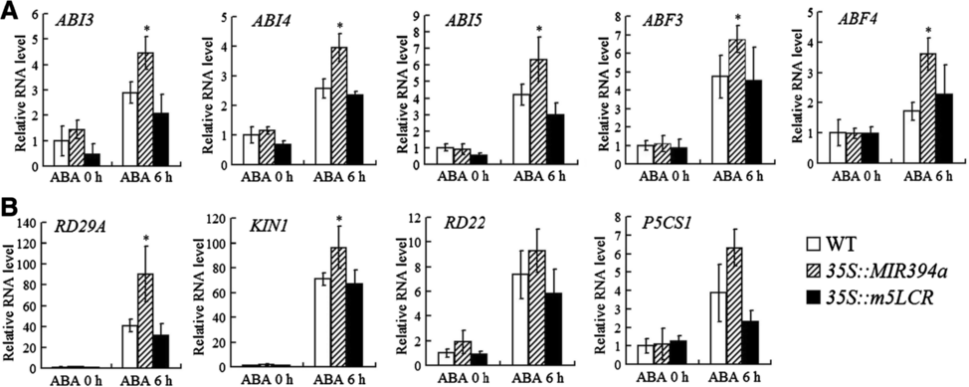
**Real-time qRT-PCR analysis of ABA-responsive genes in *35S::MIR394a *and *35S::m5LCR *plants.** Two week-old wild-type and transgenic plants were treated with 100 μM ABA for 6 h. Total RNA was isolated from the plants and analyzed by qRT-PCR using the gene-specific primers listed in Additional file 1: Table S1. The graphs indicate the induction fold of the genes with 100 μM ABA as compared with the control (0 μM ABA). For *35S:MIR394b* plants, similar results were found (data not shown). **A:** The first group genes, ABI3, ABI4, ABI5, ABF3, and ABF4; and **B:** The second group genes, RD29A, KIN1, RD22 and P5CS. Vertical bars represent standard deviation of the mean (*n* =3, treatments with 60 seedlings). Asterisks indicate the significant difference between the transgenic lines/mutants and wild-type (WT) (*P*<0.05).

To investigate whether ABA and stress-responsive genes could be regulated by miR394 or LCR over-expression, two categories of genes were tested. The first group contained five genes (*ABI3*, *ABI4*, *ABI5*, *ABF3*, and *ABF4*) coding for ABA-responsive basic leucine zipper (bZIP) transcription factors that bind to the ABA response element (ABRE) of their targets and function during the plant development and stress responses [4]. After treatment with 100 μM ABA for 6 h, all genes were induced in *35S::MIR394a* plants (Figure 10A). However, expression of *ABI3*, *ABI4*, and *ABI5* were down-regulated in *35S::m5LCR* plants.

The second group genes *RD29A*, *KIN1*, and *RD22* belong to the DRE/CRT (drought responsive/C-repeat) elements-containing class of stress- and ABA-responsive genes [28]. Another gene *P5CS* encodes enzyme D1-pyrroline-5-carboxylate synthetase involved in proline biosynthesis and ABA or abiotic stress responses [29,30]. With a similar pattern as *ABI4* or *ABI5*, all these genes *RD29A*, *KIN1*, *RD22*, and *P5CS* were induced in *35S::MIR394a* plants. By contrast, expression of *RD29A*, *KIN1*, *RD22*, and *P5CS* was down-regulated in *35S::m5LCR* plants although the degree of the gene depression varied differently (Figure 10B). These results indicated that miR394 over-expression can modify transcription of ABA- and stress-responsive genes.

## Discussion

### miR394 and LCR are involved in salt and drought stress responses

The present study identified miR394 and its target gene *LCR* involved in salt and drought stress responses in Arabidopsis. It is shown that both miR394 and LCR are independently regulated by salt and drought stresses. Because of the lower level of *LCR* expression in the presence of miR394, the partial post-transcriptional silencing of *LCR* should be responsible for the phenotypes of plants under the salt and drought stresses.

Our studies show that both miR394 and LCR are critical for plant response to salt and drought stresses. Whereas *35S:MIR394a* and *lcr* mutant plants were hypersensitive to salinity, *LCR* over-expression conferred the salt tolerance. On the other hand, *MIR394a* over-expressing plants display more tolerance to drought stress than did wild-type, whereas *35S::m5LCR* plants were hypersensitive to dehydration. These results indicate that miR394 acts as a negative regulator of plant response to salt stress but simultaneously as a positive regulator of plant tolerance to drought stress. The physiological pathway by which miR394 and LCR confers or attenuate plant tolerance to the stresses is unknown. Recently, several studies have been documented on miRNAs or small interfering RNAs (siRNAs) regulating plant responses to abiotic stresses [31]. *MIR168a* over-expressing and *ARGONAUTE1* (*AGO1*) loss-of-function *ago1-27* mutant plants are salt-hypersensitive and drought-tolerant, whereas *mir168a-2* mutant plants exhibit the reverse phenotype [13]. AGO1 is a major component of RNA-induced silencing complex (RISC) necessary for cleavage or translational block of target RNAs by loaded miRNAs [32]. Mutations in *AGO1* cause increased accumulation of miRNA targets, but as a feedback mechanism, AGO1 homeostasis itself is controlled by miR168 [33]. As miR168-regulated AGO1 mediates a broad spectrum of miRNAs that function in the RISC, it needs to study which AGO1-regulated miRNA participates in the plant response to salt and drought stresses. The present study specified the miR394 involvement in the plant abiotic stress responses. Detailed identification of the correlation between miR168 and miR394 will uncover the abiotic stress-responsive cascades.

### miR394-regulated salt and drought stress responses are dependent on ABA

The phytohormone abscisic acid (ABA) regulates numerous developmental processes and stress responses in plants. Under adverse conditions, ABA serves as an endogenous signal molecule to sense the environmental stress. Interestingly, several miRNAs and miRNA biogenesis genes have been shown to be involved in ABA-mediated stress responses [34-36]. The present study showed that miR394 were also induced by ABA treatment, although the induction was limited. To date, only a few reports are available on the genetic connection between miRNAs and ABA-mediated stress responses. A recent report shows that over-expression of gma-MIR394a confers tolerance to drought in transgenic Arabidopsis [37]. Although no LCR and ABA were mentioned and no relationship between miR394/LCR and ABA signal was identified, the result on miR394 involved in drought stress response was presented. miR159 was induced by exogenous ABA during seed germination and in young seedlings; over-expression of miR159 rendered plants hyposensitive to ABA, whereas over-expression of miR159-resistant *MYB33* and *MYB101* resulted in ABA hypersensitivity [34]. Our study has provided additional evidence that miR394 is involved in ABA or ABA-dependent salt and drought responses in Arabidopsis. First, both miR394 and pre-miR394a/b, as well as LCR were regulated by ABA and salt/drought stresses. Second, *MIR394a*-overexpressing plants displayed hypersensitivity to ABA in terms of inhibited seed germination, blocked cotyledon development, and shorter root length during the post-germination growth. Third, *MIR394a* over-expression intensified the ABA-promoted stomatal closure and generation of more ROS in the presence of ABA compared with wild-type. Furthermore, expression of ABA-responsive genes such as *ABI4*, *ABI5*, *ABF3* or *ABF4* was more evident in *35S:MIR394a* plants than in wild-type. In addition, the cross of *35S::MIR394a* with *abi4-1* or *abi5-1* mutant plants cannot rescue ABA insensitivity of *abi4-1* and *abi5-1*. These results suggest miR394-regulated salt and drought stress responses are possibly dependent on ABI4 and ABI5 and involved in the ABA response.

Recent studies have demonstrated that miRNA biogenesis genes are also involved in plant responses to abiotic stresses and ABA signaling. *HYL1* encodes a double-strand RNA-binding protein, a key factor in microRNA biogenesis [38-40]. Mutation of *HYL1* (*hyl1* mutant) resulted in ABA hypersensitivity at seed germination stage [40] (Lu and Fedoroff 2001). Furthermore, Zhang and co-workers (2008) reported that *dcl1-11* and *hen1-16* act as modulators of ABA signaling in *Arabidopsis*[35]. They found that mutation of *DCL1-11* and *HEN1-16* enhanced the ABA sensitivity of seed germination and post-germination growth by inducing ABA-responsive genes such as *ABI3*, *ABI4*, *ABI5*, *ABF3*, *KIN2* and *RD22*. From these results it can be suggested that there may be a common ABA-responsive mechanism for the phenotypes presented by *dcl1-11*, *hyl1*, *hen1-16*, *ago1-27* and *35S::MIR494a* plants. Identification of the cascades responsible for the ABA response will help to understand the networks that coordinate plant responses to ABA signal.

### LCR is a novel negative component of ABA signaling

Recently, an array of E3 proteins belonging to ubiquitin proteasome systems, are shown to actively participate in ABA hormone singling [7]. As components of E3 ubiquitin ligases of the SCF (*S*KP1-*C*ullin/*C*DC53-*F*-box) class, the F-box proteins regulate plant development and various abiotic stress responses through proteolysis system [41]. Several F-box proteins were reported to regulate ABA-dependent stress responses [30,35]. Koops and co-workers (2011) identified an Arabidopsis F-box protein EDL3 that functions as a positive regulator in ABA signal cascades controlling seed germination, root growth, and anthocyanin accumulation under abiotic stresses [42]. Arabidopsis *DOR* encodes an F-box protein (a member of the S-locus F-box-like family related to AhSLF-S2); the *DOR* null mutation caused hypersensitive ABA response of stomatal closing and improved drought tolerance; by contrast, transgenic plants over-expressing *DOR* were more susceptible to drought stress [35], indicating that F-box proteins serve as regulators of plant response to ABA-dependent abiotic stresses.

The present study identified the biological function of the F-box protein LCR. We show that LCR involves the ABA signaling in Arabidopsis under salt and drought stresses. Genetic and physiological studies revealed that while *LCR* knockdown (over-expression of miR394) or knockout (*lcr*) resulted in ABA hypersensitivity in plants with regards to the arrest of seed germination and root growth, the *LCR* over-expressing plants (*35S::m5LCR* plants) showed insensitivity to ABA. The phenotypes associated with ABA-dependent stamatal closure and water loss were also found in *35S::m5LCR* plants. Additionally, over-expression of *LCR* depressed expression of several ABA-responsive bZIP transcription factors such as *ABI3* and *ABI5*, suggesting that manipulation of *LCR* is able to modify ABA response. Notably, while miR394 expression was up-regulated by ABA, LCR was down-regulated by ABA treatment (Figure 2). This may be the result that ABA signals the inhibition of putative protein degradation through depressing LCR. On the other hand, LCR is simultaneously silenced by miR394, implying that LCR suppression may be necessary under the stress. As LCR is a novel negative regulator of ABA signaling which is able to facilitate the degradation of a putative protein, the coordinate regulation of miR394 and LCR by ABA may be essential for ABA or ABA-dependent abiotic stress responses. In our previous study, we provided the evidence that miR394 and LCR-regulated abnormal curling leaves were related to auxin signal. From the two phenotypes (leaf curling and abiotic stress response), miR394 and LCR seem to cross-talk to auxin and ABA. However, concerning a molecular mechanism, only one of them is possibly linked to miR394/LCR. Identification of targets for LCR is under the way. Further characterization of the putative component will unveil the interplay between miR394/LCR and ABA or other phytohormone signal.

## Conclusions

Both miR394 and *LCR* transcripts are regulated by salt and drought stresses and ABA treatment. The silencing of *LCR* mRNA by miR394 is essential to maintain a certain phenotype favorable for the adaptive responses to the abiotic stresses. The contrast phenotypes of ABA and abiotic stress responses may be mediated by a functional balance between miR394 and *LCR* in plants. If the balance is perturbed in case of the abiotic stress, an identical phenotype related to the stress response occurs, resulting in either ABA sensitive or insensitive response. Thus, miR394-regulated LCR abundance may allow plants to fine-tune their responses to ABA and abiotic stresses.

## Methods

### Plant materials and treatments

Arabidopsis ecotype Col-0 was used throughout the study. The *lcr* (*At1g27340*) T-DNA insertion mutants *lcr-1* (SALK_016763c) and *lcr-2* (SALK_136833c) were obtained from the Arabidopsis Biological Resource Center. Seedlings were grown in MS medium containing 1 to 3% Suc and 0.8% phytoagar (pH 5.7) or in soil (sunshine mix 5; SunGro) in a growth chamber at 22°C with 100 μE m^-2^ s^-1^ photosynthetically active radiation and a 16 h light/8 h dark cycle. Normally, two week-old seedlings were exposed to salinity (50–300 mM NaCl), drought (dehydrated on filter paper) and ABA (0.5-100 μM) for 0–12 h depending on the experiment conducted [13,30,34].

### GUS assay

Histochemical detection of GUS activity was performed using 5-bromo-4-chloro-3-indolyl β-D-glucuronic acid (X-Gluc) as a substrate. Plant tissues were placed in X-Gluc solution containing 750 mg mL^-1^ X-Gluc, 0.1% Nonidet P-40, 3 mM K_3_F_3_ (CN)_6_, 10 mM EDTA and 100 mM NaPO_4_ (pH 7) under a vacuum at room temperature for 10 min and then incubated at 37°C overnight.

### RNA gel blot analysis

Total RNA was extracted from Arabidopsis shoot and root tissues using the TRIZOL reagent (Invitrogen). Fifteen μg of total RNA was subject to a 15% denaturing polyacrylamide gel electrophoresis. The RNA on gel was transferred to the Hybond-NX membranes [43]. The membranes were hybridized with DNA oligonucleotides complementary to the miR394 sequence that was pre-labeled with γ-32P-ATP.

### Western blot analysis

Protein extracts were prepared by grinding tissues on ice in extraction buffer (5% glycerol, 4% SDS, 1% polyvinylpolypyrrolidone, 1 mM phenylmethylsulfonyl fluoride, 50 mM Tris, pH 8.0), followed by centrifugation at 4°C and 14,000 g for 15 min. 15 μg protein were separated by electrophoresis on a 12% SDS polyacrylamide gel and blotted onto polyvinylidene difluoride membranes. The immunoblot was performed with an affinity-purified LCR monoclonal antibody (1:100 dilution ), which is specifically for LCR in Arabidopsis, The horseradish peroxidase-conjugated anti-rabbit IgG was used as the secondary antibody (1:10000 dilution) [44]. The color was developed with 50 mM TBS (pH 7.6) solution containing 0.05% 3,3′-diaminobenzidine tetrahydrochloride as the horseradish peroxidase substrate. LCR antibodies were prepared by Abmart Company, Coomassie brilliant staining was used to show the equal amounts of proteins loaded.

### RT-PCR analysis

Real time-PCR and semi-quantitative PCR were performed to analyze gene transcripts based on the methods described previously [16,45]. The primers used for analysis were presented in Additional file 1: Table S1.

### Plant transformation

The plasmid construction and plant transformation were performed based on the methods described previously [16]. The target genes (precursor MIR394a/b, LRC, etc.) were PCR-amplified using primers with restriction enzyme sites at the 5'-end of forward and reverse primers, respectively (Additional file 1: Table S1). All homozygous transgenic plants (T_4_) were used in this study.

### Germination assay and root growth measurement

Seeds were surface-sterilized and grown on MS for 2 weeks under the condition of 22°C with 100 μE m^-2^ s^-1^ photosynthetically active radiation and a 16-h-light/8-h-dark photoperiod. After that they were transferred to the medium with 0–150 mM NaCl or 0–2 μM ABA for 4–10 d under the same condition. The germination (fully emerged radicle) rate was recorded and the root elongation was measured.

### Stomatal aperture measurement

Stomatal apertures were determined in the focal planes of the outer edges of guard cells in epidermal strips [46]. Detached leaves of four week-old seedlings were incubated in stomatal opening solution with 10 mM KCl, 100 mM CaCl_2_, and 10 mM MES (pH 6.1) for 2 h, and then transferred to the same solution with ABA at 0, 1, and 10 μM for 2 h. Subsequently, the adaxial surface of each leaf was applied to 3 M clear tape to peel off the epidermal layer. Epidermal strips were mounted on glass slides and observed with a microscope (YS100, Nikon, Nanjing, Jiangsu, China). Photo-graphs were taken with a Nikon digital camera (P5000 COOLPIX, Nikon, Indonesia) attached to the microscope. The ratio of width to length of the stomata was measured using Multigauge version 3.1 software (FujiFilm). More that 60 guard cells from each sample were monitored.

### Detection of reactive oxygen species

Histochemical detection of reactive oxygen species was performed based on the method described previously [47,48].

### Statistical analysis

The study was independently performed three times. Each result shown in the figures was the mean of at least three replicated treatments and each treatment contained at least 36–120 seedlings. Unless indicated, samples for analysis were randomly selected from all transgenic lines. The significant differences between treatments were statistically evaluated by standard deviation. The data between differently treated groups were further compared statistically by ANOVA followed by the least significant difference (LSD) test if the ANOVA result is significant P<0.05.

## Authors’ contributions

ZMY designed and carried out the study, and drafted the manuscript. JBS carried out transformation of genes and analysis of physiological responses. SG participated in the analysis of gene expression. HL conducted the sequence alignment. XXS participated in seedling culture and analysis of plant growth. All authors read and approved the final manuscript.

## Supplementary Material

Additional file 1: Figure S1 Transcriptional expression of pre-miR394a **(A-C)** and pre-miR394b **(D-F)** under ABA **(A, D)**, salt **(B, E)** and drought **(C, F)** treatments. Two week-old wild-type Arabidopsis seedlings were treated with NaCl (300 mM), drought, ABA (100 μM) for 0, 6 and 12 h. The expression data were obtained using quantitative real-time RT-PCR. Vertical bars represent SD of the mean three treatments (*n*=3, with 60 seedlings). Asterisks indicate that mean values are significantly different between the treatments and control (p < 0.05). **Figure S2.** Transcriptional expression of pre-miR394a **(A)** and pre-miR394b **(B)** in 35S::m5LCR transgenic lines and *lcr* mutant plants. Two week-old seedlings were used for analyzing pre-miR394a/b expression using quantitative real-time RT-PCR. Vertical bars represent SD of the mean three treatments (*n*=3, with 60 seedlings). **Figure S3.** Phenotypes of wild-type and MIM394 plants in response to ABA treatment. **A/B**: Growth of Wild-Type and MIM394 plants on MS medium, MS medium containing 0.5 μM ABA and 1 μM ABA. Seeds were germinated and grown for 4d. **C/D**: ABA dose-response analysis of germination **(C)** and cotyledon greening **(D)**. Seeds of Wild-Type and MIM394 plants were germinated for 4d on plates containing different amounts of ABA. (triplicate measurements; n = 120). **E**: Root growth measurements. Seedling root length of the indicated genotypes grown on medium containing different concentrations of ABA was measured at 7d after the end of stratification. Relative root growth compared with that on ABA-free medium is indicated. Data show the mean±SD of three replicates. At least 100 seedlings per genotype were measured in each replicate. **Table S1.** Primer and probe sequences used for this study. Click here for file
