# Supplementary material for: miR394 and LCR are involved in Arabidopsis salt and drought stress responses in an abscisic acid-dependent manner
Source: BMC Plant Biol. 2013 Dec 11;13:210. doi: 10.1186/1471-2229-13-210 (PMC3870963; doi:10.1186/1471-2229-13-210)
Supplement: Additional file 1: Figure S1 — Transcriptional expression of pre-miR394a (A-C) and pre-miR394b (D-F) under ABA (A, D), salt (B, E) and drought (C, F) treatments. Two week-old wild-type Arabidopsis seedlings were treated with NaCl (300 mM), drought, ABA (100 μM) for 0, 6 and 12 h. The expression data were obtained using quantitative real-time RT-PCR. Vertical bars represent SD of the mean three treatments (n=3, with 60 seedlings). Asterisks indicate that mean values are significantly different between the treatments and control (p < 0.05). Figure S2. Transcriptional expression of pre-miR394a (A) and pre-miR394b (B) in 35S::m5LCR transgenic lines and lcr mutant plants. Two week-old seedlings were used for analyzing pre-miR394a/b expression using quantitative real-time RT-PCR. Vertical bars represent SD of the mean three treatments (n=3, with 60 seedlings). Figure S3. Phenotypes of wild-type and MIM394 plants in response to ABA treatment. A/B: Growth of Wild-Type and MIM394 plants on MS medium, MS medium containing 0.5 μM ABA and 1 μM ABA. Seeds were germinated and grown for 4d. C/D: ABA dose-response analysis of germination (C) and cotyledon greening (D). Seeds of Wild-Type and MIM394 plants were germinated for 4d on plates containing different amounts of ABA. (triplicate measurements; n = 120). E: Root growth measurements. Seedling root length of the indicated genotypes grown on medium containing different concentrations of ABA was measured at 7d after the end of stratification. Relative root growth compared with that on ABA-free medium is indicated. Data show the mean±SD of three replicates. At least 100 seedlings per genotype were measured in each replicate. Table S1. Primer and probe sequences used for this study. [file 1471-2229-13-210-S1.doc]

**Additional file 1: Figure S1. Transcriptional expression of pre-miR394a (A-C) and pre-miR394b (D-F) under ABA (A, D), salt (B, E) and drought (C, F) treatments.** Two week-old wild-type Arabidopsis seedlings were treated with NaCl (300 mM), drought, ABA (100 μM) for 0, 6 and 12 h. The expression data were obtained using quantitative real-time RT-PCR. Vertical bars represent SD of the mean three treatments (*n*=3, with 60 seedlings). Asterisks indicate that mean values are significantly different between the treatments and control (p < 0.05).

**Additional file 1: Figure S2. Transcriptional expression of pre-miR394a (A) and pre-miR394b (B) in 35S::m5LCR transgenic lines and *lcr* mutant plants.** Two week-old seedlings were used for analyzing pre-miR394a/b expression using quantitative real-time RT-PCR. Vertical bars represent SD of the mean three treatments (*n*=3, with 60 seedlings).

**
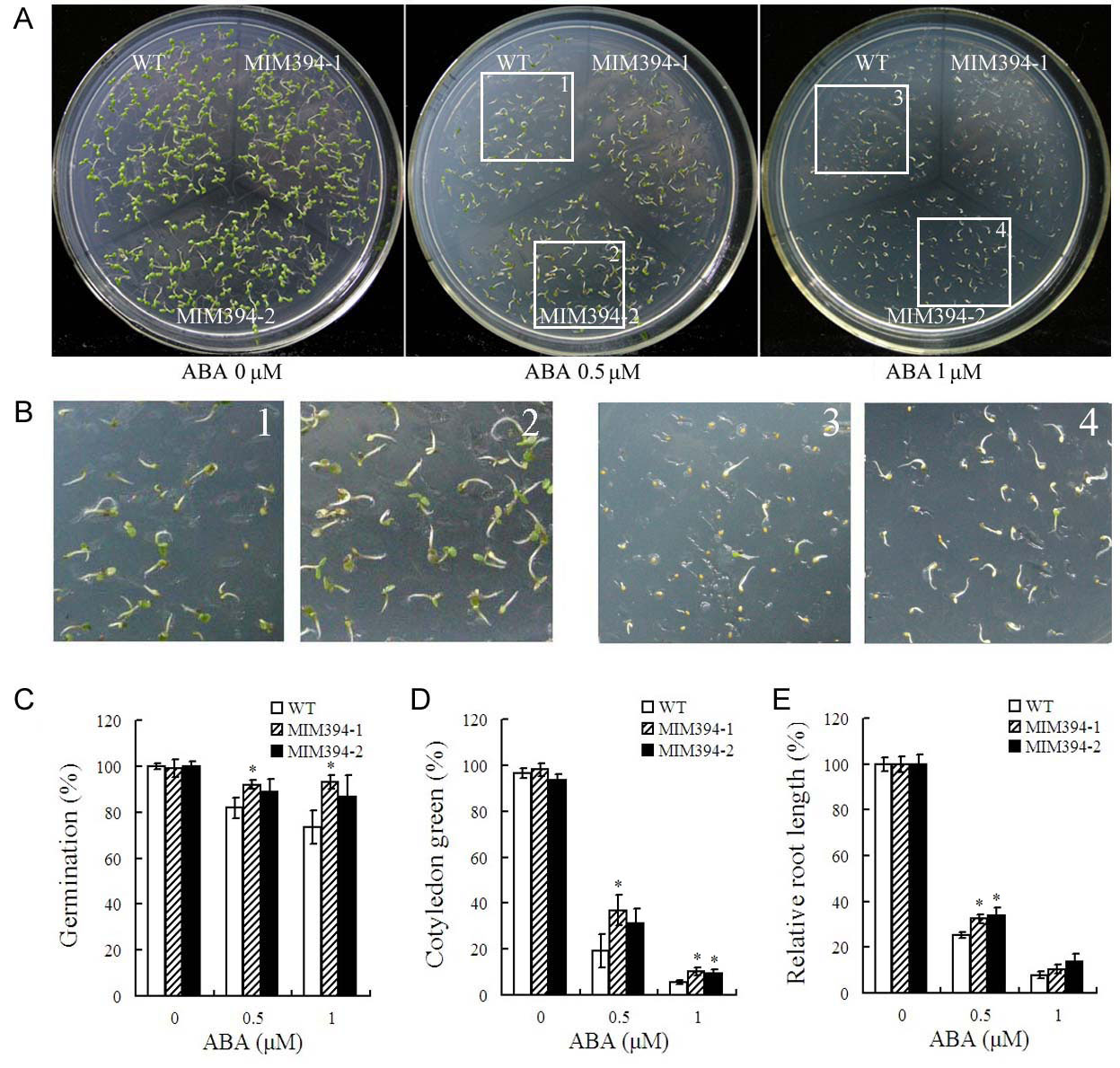
**

**Additional file 1:Figure S3. Phenotypes of wild-type and MIM394 plants in response to ABA treatment.**

A/B: Growth of Wild-Type and MIM394 plants on MS medium, MS medium containing 0.5 μM ABA and 1 μM ABA. Seeds were germinated and grown for 4d.

C/D: ABA dose-response analysis of germination (C) and cotyledon greening (D). Seeds of Wild-Type and MIM394 plants were germinated for 4d on plates containing different amounts of ABA. (triplicate measurements; n = 120).

E: Root growth measurements. Seedling root length of the indicated genotypes grown on medium containing different concentrations of ABA was measured at 7d after the end of stratification. Relative root growth compared with that on ABA-free medium is indicated. Data show the mean±SD of three replicates. At least 100 seedlings per genotype were measured in each replicate.

**Additional file 1: Table S1. Primer and probe sequences used for this study**

|  | Primer name | Forward (5'-3') | Reverse (5'-3') |
| --- | --- | --- | --- |
| Genotyping PCR | P1 and P3 | TATTGTCTTGATACCAA | GCTTCATCTTTTTCCATGGT |
|  | P2 and P4 | TGCCATGCTTAGACAGC | CTACCGCAACACGAGAC |
|  | LB1.3 | TCAAACAGGATTTTCGCCTGCT |  |
| Northern blot | LCR-Probe | GGAGGTGGACAGAATGCCAA |  |
| RT-PCR | LCR-RT | CCCGTCCAACATAAAGC | GCAAGCGATCCAAAGTC |
|  | Pre-miR394a-RT | AATCTATCGCAAACTCC | TCGCCAAGAAACAAATC |
|  | Pre-miR394b-RT | ACAGAAAGGAAATGAGTG | TGAGGGGTTTTACAAAG |
| qRT-PCR | Pre-miR394a-RT |  |  |
|  | Pre-miR394b-RT |  |  |
|  | LCR-qRT | CTCTGGGAAATGGAAACA | TCATCTGTCTGGACCTCAA |
|  | GUS-qRT | TCAGTGGCAGTGAAGGG | GAGGTACGGTAGGAGTTGG |
|  | ABI3-qRT | GTGGTCGCTTCACCAACTTCTC | CAGCTTTAATCATGACCCTCCA |
|  | ABI4-qRT | TTAGGGCAGGAACAAGG | TCCAGACCCATAGAACATAC |
|  | ABI5-qRT | GGAGATTGCGGACATTGATGAG | GGGAACACTAGTAAAGCAGATC |
|  | ABF3-qRT | TGGAAAAGCAGAAAAATCAGC | CAAGCATTGCCTTTTGCAT |
|  | ABF4-qRT | ACTGGAAGCCGAAATTGAAAAGCTC | CACCATGGTCCGGTTAATGTCCT |
|  | RD29A-qRT | GTTACTGATCCCACCAAAGAAGA | GGAGACTCATCAGTCACTTCCA |
|  | P5CS-qRT | AGCAGCCTGTAATGCGATGG | AAGTGACGCCTTTGGTTTGC |
|  | KIN1-qRT | TGGAGCTGGAGCACAACA | GACCCGAATCGCTACTTGTTC |
|  | RD22-qRT | AGGGCTGTTTCCACTGAGG | CACCACAGATTTATCGTCAGACA |
|  | COR15A-qRT | GCTTCAGATTTCGTGACGGATAAAAC | GCAAAACATTAAAGAATGTGACGGTG |
|  | ACTIN | CATCAGGAAGGACTTGTACGG | GATGGACCTGACTCGTCATAC |
| Construction | ProLCR::GUS | CCGGAATTCTATTGTCTTGATACCAA | GGACTAGTGCTTCATCTTTTTCCATGGT |
|  | 35S::miR394a | catgccatggccgtcataaagagaac  tcatctgcc | gactagtttcatcgccaagaaacaaatc |
|  | LCR-CDS | GAAGATCTATGGAAGAAGAGCTTGCC  ATG | GACTAGTATGGAAGAAGAGCTTGCCATG |
|  | m5LCR | GCACCATATGTTCGGCATGCGATCAAC  TTC CTTCCACAACAGTGT | ACACTGTTGTGGAAGGAAGTTGATC  GCA TGCCGAACATATGGTGC |
|  | IPS1-CDS | GAAGATCTaagaaaaatggccatccc  ctagc | GGACTAGTGAGGAATTCACTATAAA  GAGAATCG |
|  | MIM394 | aattTGGCATTCaccaGTCCACCTCCa  gcttcggttcccctcg | CTGGAGGTGGACTAGAGAATGCCAA  ATTTCTAGAGGGAGATAA |
